# Can Haematological Parameters Discriminate COVID-19 from Influenza?

**DOI:** 10.3390/jcm13010186

**Published:** 2023-12-28

**Authors:** Sahar Gnaba, Dmitry Sukhachev, Tiffany Pascreau, Félix Ackermann, Frédérique Delcominette, Florence Habarou, Aurélie Védrenne, Emilie Jolly, Elena Sukhacheva, Eric Farfour, Marc Vasse

**Affiliations:** 1 Biology Department, Foch Hospital, 92150 Suresnes, France; gnabasahar@gmail.com (S.G.); t.pascreau@hopital-foch.com (T.P.); f.delcominette@hopital-foch.com (F.D.); f.habaraou@hopital-foch.com (F.H.); a.vedrenne@hopital-foch.com (A.V.); e.jolly@hopital-foch.com (E.J.); e.farfour@hopital-foch.com (E.F.); 2 LabTech Ltd., Saint-Petersburg 195027, Russia; dmitry.sukhachev@gmail.com; 3 INSERM Hémostase Inflammation Thrombose HITh U1176, Université Paris-Saclay, 94276 Le Kremlin-Bicêtre, France; 4 Department of Internal Medicine, Foch Hospital, 92150 Suresnes, France; f.ackermann@hopital-foch.com; 5 Beckman Coulter Eurocenter, 1260 Nyon, Switzerland; esukhacheva@beckman.com

**Keywords:** COVID-19, influenza, respiratory syncytial virus, complete blood count, cellular population data, B-lymphocytes, CD16^pos^ monocytes, logistic regression model

## Abstract

Symptoms of COVID-19 are similar to the influenza virus, but because treatments and prognoses are different, it is important to accurately and rapidly differentiate these diseases. The aim of this study was to evaluate whether the analysis of complete blood count (CBC), including cellular population (CPD) data of leukocytes and automated flow cytometry analysis, could discriminate these pathologies. In total, 350 patients with COVID-19 and 102 patients with influenza were included between September 2021 and April 2022 in the tertiary hospital of Suresnes (France). Platelets were lower in patients with influenza than in patients with COVID-19, whereas the CD16^pos^ monocyte count and the ratio of the CD16^pos^ monocytes/total monocyte count were higher. Significant differences were observed for 9/56 CPD of COVID-19 and flu patients. A logistic regression model with 17 parameters, including among them 11 CPD, the haemoglobin level, the haematocrit, the red cell distribution width, and B-lymphocyte and CD16^pos^ monocyte levels, discriminates COVID-19 patients from flu patients. The sensitivity and efficiency of the model were 96.2 and 86.6%, respectively, with an area under the curve of 0.862. Classical parameters of CBC are very similar among the three infections, but CPD, CD16^pos^ monocytes, and B-lymphocyte levels can discriminate patients with COVID-19.

## 1. Introduction

During the first winter of the SARS-CoV-2 pandemic (from December 2019 to March 2020), the number of flu patients was very low in France [1] and in other countries [2,3]. However, in the following years, even though COVID-19 was the most common respiratory viral pathology, cases of influenza and respiratory syncytial virus (RSV) infections have reappeared [4].

The presentation of COVID-19 is similar to other respiratory infections, but as the treatments and prognoses of COVID-19 and other respiratory viruses are different, it is important to accurately differentiate these different respiratory tract infections. The gold standard for an acute diagnosis of respiratory viral infection is, of course, polymerase chain reaction (PCR) on a nasal swab, but the time for carrying out the PCR can be long, especially in the event of a massive influx of patients in the emergency department (ED) during the winter period. Meanwhile, these patients require isolation measures to avoid contagion. They are also expensive. A few studies have attempted to identify significant differences between classical haematological and biochemical parameters in patients with influenza and COVID-19. The most frequent findings were significant differences between absolute monocyte counts [5,6,7] and ratios, including neutrophils, monocytes, or platelets [8,9,10,11]. However, the differences, although statistically significant, are small and would not suffice to distinguish between the illnesses in the ED [12].

The white blood cell (WBC) differential analysis performed on Beckman Coulter haematology analysers is based on “VCS technology”. Briefly, each leukocyte is characterized by its volume “V” (measured through direct impedance), its conductivity “C” (analyzed through conductivity in radio frequency current), and its scatter “S” of a laser beam. Based on these three parameters, the analyses identify the five subpopulations present in normal blood and calculate for each subpopulation the average and the standard deviation of the different cell subtypes. It has been shown that VCS parameters, also called “cellular population data” (@CPD), can discriminate patients with positive SARS-CoV-2 PCR from patients with negative SARS-CoV-2 PCR [13,14].

Therefore, the aim of this study was to evaluate whether CPD, including not only Monocyte Distribution Widths (MDWs), which are CE-marked, can be an aid to identify COVID-19 patients in the ED) could be useful to discriminate COVID-19 from the flu. Indeed, a quick and inexpensive way to discriminate between patients with COVID and the flu would make it possible to possibly initiate a specific treatment for each of these two diseases, because the best results from antivirals are obtained when the treatment is started early. As variations in lymphocyte and monocyte subpopulations in the flu and COVID-19 have been reported [15,16,17], we also evaluated whether their consideration could be interesting for this discrimination using a commercial flow cytometry assay (CytoDiff™). CytoDiff™ is used for flow leukocyte differential and, in addition to five-part diff, it is able to enumerate subpopulations of immune cells (specifically, lymphocyte and monocyte subpopulations). Our results show that consideration of CPD alone is not sufficient to discriminate these pathologies, but the consideration CD16^pos^ monocyte and B-lymphocyte levels can greatly help to identify patients with COVID-19.

## 2. Materials and Methods

### 2.1. Study Population

This study was performed between 14 September 2021 and 24 April 2022. Patients were included consecutively if they had a positive RT-qPCR for SARS-CoV-2 or for influenza (A or B) associated with a blood count. Patients were discarded if they were pregnant, if they were under immunosuppressive agents for organ transplantation, if they were treated with corticosteroids due to chronic inflammatory or auto-immune diseases, if they were treated with antineoplastic therapy within the last month before viral infection diagnosis, or if they had a haematological disease (lymphoproliferative disease or myelodysplastic syndrome). Simultaneous positivity for two viral diseases was also a criterion of exclusion.

**Figure 1:**
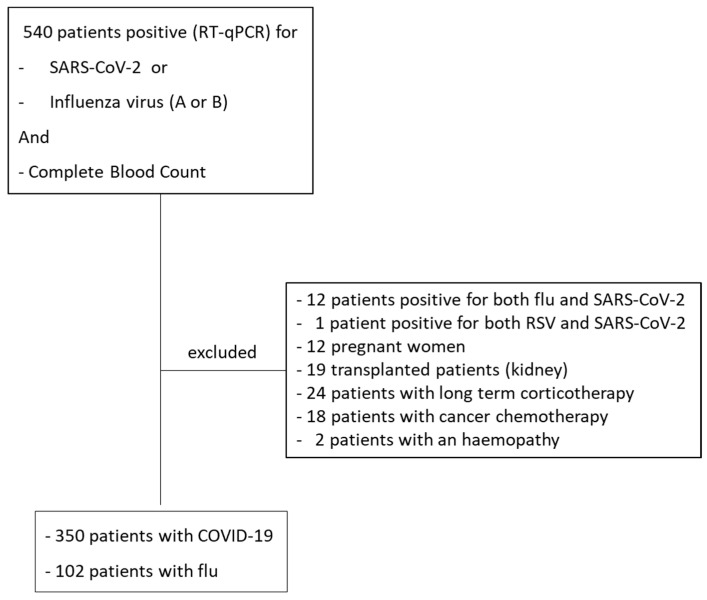
Flow-chart of the inclusion of the patients from 14 September 2021 to 24 April 2022.

Figure 1 presents a diagram of the flow of the patients included in this study. Finally, 350 patients with COVID-19 and 102 patients with flu were included. A group of 177 subjects with a normal CBC and differential referred to our institution from 11 October 2021 to 18 October 2021 without evidence of infectious diseases was used as controls to define the usual values of CPD.

### 2.2. WBC Count and Differential

Blood was collected in 3 mL S-Monovettes (Sarstedt, Marnay, France) containing 4.8 mg EDTA-K3. CBC analyses were performed within 6 h after collection using the DxH 800 analyser (Beckman Coulter, Inc., Brea, CA, USA). WBC differential analysis was performed using the Coulter DxH 800 analyser was based on the measurement of seven distinct parameters for each cellular event; in addition to the volume (V) and conductivity (C), there are different measurements of the scatter, as previously described [18]. Currently, these morphometric parameters are for research use only. Some patients, particularly in the COVID-19 group, had very low levels of eosinophils, and therefore, for those patients, @CPD-EO were missing in 40 cases for COVID-19 patients and 7 cases for the flu.

A differential was performed within 24 h using the CytoDiff™ reagent (Beckman Coulter) according to the manufacturer’s instructions. CytoDiff™ analysis, based on the study of Faucher et al. [19], was based on a pre-mixed cocktail of six antibodies with five colours (CD36-FITC, CD2-PE, CRTH2-PE, CD19-ECD, CD16-PC5, and CD45-PC7), and it for allowed for the detection and quantification of 13 populations of WBC thanks to an exclusion gating strategy among WBC and an autogating software that allows for getting rid of any operator intervention [20]. A biparametric histogram (CD16/SSC) conditioned on the monocyte population distinguishes CD16^pos^ from classical monocytes, which are CD16-negative. Direct immuno-labelling was performed on 100 µL of whole peripheral EDTA-anticoagulated blood using a TQ-PREP (Beckman-Coulter) according to the instructions of the manufacturer. CytoDiff™ was systematically performed on patient samples during the week, but not during the weekend (Saturday and Sunday), and therefore it was available only for 276 patients with COVID-19 (78.9%) and for 59 patients with the flu (56.2%).

### 2.3. Other Biological Parameter Measurements

Diagnosis of SARS-CoV-2 and Influenza (A and B) was made through RT-qPCR based on a nasopharyngeal swab using the Alinity M RESP-4-Plex RT-PCR assay (Abbott Molecular, Des Plaines, IL, USA). C-reactive protein (CRP) was measured using Alinity IC (Abbott Diagnostics, Rungis, France), D-dimers (D-dimers HS 500 IL) were quantified using the ACL TOP analysers (Werfen, le Pré-Saint-Gervais, France), and the blood gas was analysed using GEM 4000 (Werfen).

### 2.4. Statistical Analyses

As most of the parameters tested were non-normally distributed, the results were expressed as the median and the 25th and 75th percentiles. Statistical analyses were performed using MedCalc software, version 19.2 (MedCalc Software Ltd., Ostend, Belgium). The chi-square test was used for comparisons of frequencies, the Kruskal–Wallis test and Dunn’s post hoc tests were employed for multiple group comparisons, and the Mann–Whitney test was used for comparisons of 2 groups. The performance of individual parameters to discriminate different subgroups of patients was analyzed using receiver operating characteristic (ROC) analysis and the area under the curve (AUC). A parameter was considered to have a discriminating potential when the AUC was above 0.7. Categorical variables were compared using the chi-squared test. To develop the algorithm to differentiate patients with COVID-19 from patients with the flu, we considered the patients with the flu as the negative group and patients with COVID-19 as the positive group. All DxH 800 or CytoDiff™ parameters were first included in the model, and then we excluded parameters according their *p*-value (the significance value of an individual parameter into the logistic regression model), with the highest value first. We excluded one parameter at each step, as *p*-values of the remaining parameters are changed in the updated model at each step. The termination criteria of the process were not low *p*-values for all parameters, as recommended by MedCalc, but rather the balance between false-negative and false-positive results when we could not further decrease those numbers. The number of false-negative/false-positive results were calculated at each step of the backward procedure. Finally, the cut-off of the model was adjusted to achieve a clinically meaningful balance between false-positive and false-negative results.

## 3. Results

### 3.1. Characteristics of the Population

Patients with the flu were slightly younger than patients with COVID-19. Regarding clinical signs, patients with influenza were more frequently febrile than patients with COVID-19, had a cough more frequently, and were less frequently hospitalized than patients with COVID-19 (Table 1).

Concerning biological data, CRP and O_2_ saturation were significantly higher in patients with the flu than in patients with COVID-19 (Table 1), as well as the CD16^pos^ monocyte count and the CD16^pos^ monocyte-to-monocyte count ratio, and patients with the flu had a lower platelet count than patients with COVID-19 (Table 2). Different ratios, such as the lymphocyte-to-monocyte ratio or the platelet-to-lymphocyte ratio, are not reported in the table because they were not statistically different between the different groups of patients.

### 3.2. Variations in CPD According to the Pathology

For the different pathologies, the CPD of the different blood lineages were, most often, different from the CPD of the controls (Table S1). For COVID-19 patients, 41/56 CPD (73.2%) were different (5/14 (35.7%) for NE, 14/14 (100%) for LY and MO, and 8/14 (57.1%) for EO), and for the flu, 39/56 (69.6%) were different (6/14 (42.9%) for NE, 14/14 (100%) for LY, 13/14 (92.8%) for MO, and 6/14 (42.8%) for EO). However, if we consider differences between CPD for both pathologies, significant differences were only observed for 9/56 (16.1%) CPD (Table 3). If we compare COVID-19 and the flu, only nine CPD were different between these pathologies, with three concerning neutrophils, five concerning monocytes, and one concerning eosinophils.

### 3.3. Potential for CPD and Other Parameters of the Blood Cell Count to Discriminate Patients with the Flu from Patients with COVID-19

Lastly, we tried to analyse whether CBC parameters, including CPD, could contribute to discriminating patients with the flu from patients with COVID-19. As indicated above, platelet count and nine CPD were significantly different between patents with COVID-19 and the flu, but when we performed the ROC curve analysis, the highest AUC was 0.603 for SD-V-MO, and we determined that none of these parameters alone was effective for discriminating patients with COVID-19 from patients with the flu. When we considered the results of the CytoDiff™, CD16^pos^ monocytes and the ratio of CD16^pos^ monocytes/total monocytes were the most discriminant, with cut-offs of 0.064 × 10^9^/L and 0.017 but AUCs of 0.612 and 0.672, respectively. Therefore, we used logistic regressions to try to discriminate both groups of patients.

The first model included only parameters from DxH 800 and included 16 CPD (4 CPD of neutrophils (@SD-MALS-NE, SD-UMALS-NE, MN-LMALS-NE, and SD-LAMS-NE), 5 CPD of lymphocytes (@MN-C-LY, @MN-MAL-LY, @MN-UMALS-LY, @MN-LMALS-LY, and @MN-LALS-LY), 4 CPD of monocytes (@SD-V-MO, @SD-MALS-MO, @SD-UMALS-MO, and @MN-AL2-MO), and 3 CPD of eosinophils (@MN-MALS-EO, @SD-LMALS-EO, and @SD-LALS-EO)), the haematocrit, the mean corpuscular haemoglobin concentration, and the RDW. The AUC for the regression model was 0.777. To improve the performance, we used another logistic regression including 17 parameters, among them 11 CPD (5 CPD of lymphocytes (@MN-V-LY, @MN-MAL-LY, @MN-UMALS-LY, @MN-LMALS-LY, and @MN-AL2-LY), 4 CPD of monocytes (@MN-V-MO, @SD-MALS-MO, @MN-LMALS-MO, and @MN-LALS-MO), and 2 CPD of eosinophils (@MN-LALS-EO and @SD-LALS-EO)), the haemoglobin level, the haematocrit, the RDW, and three parameters from the CytoDiff™ (the absolute count of B-lymphocytes and DC16^pos^ monocytes and the ratio of CD16^pos^ monocytes/total monocytes). The AUC for this regression model was 0.862 (95% CI 0.817–0.899). With a cut-off of 0.5, the sensitivity and efficiency of the model were 96.2 and 86.6%, respectively (Table 4).

## 4. Discussion

Over the past three years, a very large number of articles have been devoted to the description of the clinical signs and biological abnormalities associated with SARS-CoV-2 infection. It is now clear that COVID-19 is an endemic disease that can saturate emergency services, especially when viral respiratory pathologies are present in large quantities, such as influenza or RSV infection, as we observed in December 2021 in France. A large Swiss study showed that prognoses of in-hospital COVID-19 and flu patients were significantly different: the mortality of patients with COVID-19 was significantly higher than in patients with the flu, the hazards of ICU admission and in-hospital death were about two-fold to three-fold higher for COVID-19 patients than for flu patients, and the hospital stay was longer for COVID-19 [21,22]. Additionally, the clinical course is quite different between both diseases, because with influenza, strong inflammation associated with a “cytokine storm” is relatively rare. Unlike COVID-19, treatments are quite different. For the treatment of influenza, many anti-influenza drugs have been developed that target the virus itself, while treatment of COVID-19 aims to avoid this exaggerated inflammatory reaction and the associated thromboembolic complications. For flu treatment, it has been observed that there was an increase in the mortality hazard rate with each day’s delay in initiation of treatment up to day five compared with treatment initiated within two days of symptom onset. Therefore, the faster the diagnosis is made, the more effective the treatment will be [23].

The gold standard for the diagnosis of respiratory viruses is RT-PCR. However, RT-PCR is time-consuming, it requires trained technicians, and rapid tests are expensive. Additionally, because of huge influxes of patients at the same time in the ED of cases of high viral circulation, laboratory departments can be overwhelmed with this massive activity. The absence of medical workers, who were affected themselves by the disease, led to a delay in the diagnosis and triage of the patients. Therefore, a less expensive test that could easily and quickly discriminate the type of virus involved in the patients’ clinical symptoms is welcome. CBC is usually prescribed in most ED patients with fever and cough. We previously described that the combination of four CPD could identify patients with COVID-19 [13]. Among these CPD, the elevation of SD-V-MO (or Monocyte Distribution Width—MDW for DxH900) was shown to be a useful marker, which is CE-marked, to identify patients with COVID-19 [24,25]. In this paper, we show that the increase in SD-V-MO is observed not only in COVID-19, but also in the flu and in RSV infection. In agreement with this report, two other studies also observed an increased level of SD-V-MO in the flu [26,27]. Therefore, MDW (or SD-V-MO) alone cannot help to discriminate flu from COVID-19 patients. We also studied the CPD from the different lineages; the vast majority of them were different from controls, but only nine of them were significantly different between patients with the flu and COVID-19, and using ROC curve analysis, none of them alone were discriminant to distinguish both pathologies. Kala et al. also studied CPD in patients with COVID-19 and “non-COVID influenza-like illness” [28]. In contrast to our study, they only considered the mean levels (MNs) but not the standard deviations (SDs) of CPD, and they did not identify the type of virus for patients negative for COVID-19; therefore, their population, called “Non-COVID Influenza-like Illnesses”, is heterogeneous. This could explain why they observed a higher number of CPD that differed between their two groups of patients. For example, they observed that 6/7 neutrophil CPD were different between patients with COVID-19 and patients without COVID-19. However, as in our study, they observed small variations, which cannot be used to discriminate patients with COVID-19 infection from patients with other pulmonary pathogens. Similarly to our results, they observed the most significant differences with the MN-V-NE and the MN-V-MO, indicating an active participation of these cells in inflammatory mechanisms specific to SARS-CoV-2 infection. Analysis of differential absolute counts (as well as different ratios derived from these counts, such as the neutrophil-to-lymphocyte ratio (NLR), lymphocyte to monocyte ratio (LMR), or platelet-to-lymphocyte ratio (PLR)) did not reveal any significant differences between the different cellular types. In agreement with our study, Lin et al. [26] and Badaki-Makun et al. [27] did not observe any differences for NLR between patients with the flu and COVID-19, whereas two studies suggested that NLR could be discriminant, and patients with the flu had a higher NLR than patients with COVID-19 [8,9]. These differences could be due to different degrees of severity of respiratory diseases and the sizes of the populations recruited. Our study comparing these two groups of patients was the largest because data from 350 patients with COVID-19 and 103 patients with the flu were analyzed, whereas for the four others studies, the number of patients with COVID-19 varied between 9 [26] and 120 [9], and the number varied from 24 [26] to 100 [9] for patients with the flu. In addition, for the two studies describing a significant difference in NLR for patients with the flu or COVID-19, the comparison was performed with patients with the flu enrolled from 2018 to 2019 [8,9], whereas in our study and the studies from Lin et al. [26] and Badaki-Makun et al. [27], patients with COVID-19 and the flu were prospectively included either in 2020–2021 or 2021–2022. We cannot exclude that the different strains of virus induced different biological modifications.

Circulating monocytes participated in all stages of SARS-CoV-2 infection [29], and their absolute count varied according to the severity of the disease, with variations in classical and “non-classical” (or “inflammatory”) monocyte levels [17,30,31,32]. Contrasting results were published concerning the differences in absolute monocyte counts in COVID-19 and the flu. Curtolo et al. [5] described a significantly higher level of monocytes in COVID-19 patients than in patients with the flu, whereas Chen et al. described the opposite [6]. Similarly to Kazancioglu et al. [9] and Luo et al. [33], we did not observe any significant differences between the two groups of patients. These differences could be due to the severity of the disease in the patients, as it was previously observed that monocyte levels decreased with the severity of COVID-19 [17,34]. However, we observed a significant difference between COVID-19 and flu patients for non-classical (CD16^pos^) monocytes, as higher levels of CD16^pos^ monocytes were observed in patients with the flu. To our knowledge, monocyte subpopulations are rarely studied in influenza, but in agreement with our study, an increase in non-classical monocytes in flu patients had already been observed [15,16]. Therefore, the ratio of CD16^pos^ monocytes/monocytes had the most potential to discriminate patients with the flu from those with COVID-19. However, the AUC of the ROC curve did not reach the cut-off of 0.7 that is usually considered to be useful for relevant diagnosis in biology. Consequently, we used a logistic regression model using CPD with and without CytoDiff™ results to identify patients with COVID-19 from flu patients. We favoured a model that allowed us to specifically identify patients with COVID-19 due to the specificity of care for this pathology. Using the parameters of the CBC (including the CPD) and two parameters of the CytoDiff™ analysis, this algorithm had an efficiency of 86.6%, and it could provide interesting information concerning the risk of COVID-19. A model to discriminate patients with COVID-19 from patients with the flu using biological parameters has already been proposed, and it was based on 18 parameters, including haematology and biochemical and coagulation tests [18]. Our model has an advantage in that it requires only the parameters of the CBC and it is less expensive than a model that requires different types of biological markers. All of the parameters needed for our model can be obtained using only one EDTA sample, which limits the multiplication of samples and laboratory congestion by reducing the number of tubes handled.

Our study has some limitations: it is a single-centre experience with a limited number of patients in each group. This limited us to only investigating differences between influenza and COVID-19. In addition, as the influenza virus changes every year, we do not know whether the different strands of the virus induced similar variations in CPD. However, for SARS-CoV-2, despite different mutations since the beginning of the pandemic, similar variations in CPD were observed with the original virus [13], and the Omicron virus was the most represented during this study in 2022.

## 5. Conclusions

In conclusion, our study indicated that similar variations in the CPD of patients with COVID-19 or influenza were present. Therefore, these parameters alone were not sufficient to discriminate these pathologies. However, consideration of the levels of CD16^pos^ monocytes, as well as B-lymphocytes levels, can greatly help to identify patients with COVID-19. The use of such algorithms requires the integration of computers into the analysers or in the laboratory information system to provide diagnostic guidance.

## Figures and Tables

**Table 1:** Main clinical and biological characteristics of patients presenting with COVID-19 or the flu at the emergency department.

	SARS-CoV-2 *n* = 350	Influenza Virus *n* = 102	*p*-Values
**Females/males**	(160/190)	(56/46)	0.1275
**Age** (years) (25th–75th percentile)	66	60	0.0247
	(0–82)	(39–73)	
**Symptoms**, n (*%*)			
*Fever* (>38 °C)	46 (13.1)	27 (26.5) °°	0.0020
*Chills*	28 (8)	15 (14.7)	0.0661
*Cough*	118 (33.7)	62 (60.8) °°°	<0.0001
*Dyspnoea*	72 (20.6)	16 (15.7)	0.3385
*Headache*	72 (20.6)	28 (27.5)	0.1789
*Nausea*	71 (20.3)	24 (23.5)	0.5756
*Diarrhea*	44 (12.6)	9 (8.8)	0.3820
*Myalgia*	76 (21.7)	23 (22.5)	0.9714
**Follow-up**, n (%)			
*Discharged*	204 (58.3)	83 (81.4) °°°	<0.0001
*Hospitalized*	138 (39.4)	18 (17.6) °°°	0.0001
*ICU*	8 (2.3)	1 (1)	0.0001
**Specific PCR (Ct)**	20.1	23.0	N.D
	(16.9–26.1)	(18.0–28.0)	
**pH**	7.46	7.44	0.5711
	(7.43–7.48)	(7.41–7.48)	
**O_2_ saturation (%)**	97	98 °	0.0293
	(95–99)	(96–99)	
**CRP (mg/L)**	28.1	41.7 °	0.0299
	(6.8–79.4)	(16.8–76.2)	
**D-dimers (mg/L)**	0.693	0.651	0.8104
	(0.368–1.122)	(0.349–1.261)	

CRP: C-reactive protein; ICU: intensive care unit; ND: not done. Values are expressed as median and 25th percentile–75th percentile. The chi-square test was used for comparisons of frequencies and the Mann–Whitney test was employed for group comparisons. ° *p* < 0.05, °° *p* < 0.01, °°° *p* < 0.001 versus COVID-19.

**Table 2:** Blood parameters of patients presenting with COVID-19 or the flu at the emergency department.

	SARS-CoV-2	Influenza Virus	*p*-Values
**White blood cells** (×10^9^/L)	6.5	6.50	0.6325
	(5.1–8.4)	(5.2–8.3)	
*Neutrophils* (×10^9^/L)	4.6	4.7	0.4616
	(3.2–6.5)	(3.6–6.1)	
*Eosinophils* (×10^9^/L)	0.0	0.0	0.3247
	(0.0–0.1)	(0.0–0.0)	
*Total lymphocytes* (×10^9^/L)	1.0	0.9	0.1326
	(0.7–1.5)	(0.6–1.4)	
*B-lymphocytes* (×10^9^/L)	0.122	0.085	0.0943
	(0.070–0.189)	(0.053–0.156)	
*T-lymphocytes* (×10^9^/L)	0.783	0.604	0.1184
	(0.478–1.213)	(0.372–1.096)	
*NK-lymphocytes* (×10^9^/L)	0.213	0.188	0.1184
	(0.132–0.346)	(0.118–0.298)	
*Monocytes* (×10^9^/L)	0.6	0.7	0.3765
	(0.4–0.9)	(0.5–0.9)	
*CD16^pos^ monocytes* (×10^9^/L)	0.087	0.146 °°	0.0063
	(0.040–0.195)	(0.079–0.204)	
*Ratio neutrophils/lymphocytes*	4.40	5.00	0.2580
	(2.41–8.28)	(2.89–9.13)	
*Ratio of CD16^pos^ monocytes/monocytes*	0.15	0.24 °°°	0.0006
	(0.09–0.25)	(0.16–0.33)	
**Haemoglobin (g/dL)**	13.5	13.2	0.4114
	(12.3–14.5)	(12.3–14.4)	
*RDW*	13.7	13.7	0.6286
	(13.2–14.6)	(13.2–14.4)	
**Platelets (×10^9^/L)**	217	197 °°	0.0045
	(176–273)	(162–234)	

ND: not done; RDW: red blood cell distribution width. Values are expressed as median and 25th percentile–75th percentile. The Mann–Whitney test was employed for group comparisons. °° *p* < 0.01, °°° *p* < 0.001 versus COVID-19.

**Table 3:** Comparisons of cellular population data (CPD) of 178 controls (C) with CPD of 349 patients with proven COVID-19 and 102 patients with the flu.

	Controls	SARS-CoV-2	Influenza Virus	*p*-Values
@MN-V-NE	147	149 **	152 ***^/^°°	<0.0001
	(144–152)	(145–155)	(148–156)	
@SD-V-NE	17.1	17.8 ***	18.1 ***^/^°	<0.0001
	(16.3–17.8)	(16.9–19)	(17.3–19.1)	
@SD-C-NE	4.5	4.8 ***	4.9 ***^/^°	<0.0001
	(4.2–4.9)	(4.4–5.2)	(4.6–5.3)	
@MN-V-MO	176	183 ***	187 ***^/^°°	<0.0001
	(172–179)	(177–191)	(181–193)	
@SD-V-MO	18.8	23.7 ***	24.4 ***^/^°°	<0.0001
	(17.7–19.6)	(21.5–25.3)	(22.8–26.1)	
@MN-MALS-MO	88	86 ***	85 ***^/^°	<0.0001
	(86–90)	(83–89)	(82–88)	
@MN-LMALS-MO	75	73 ***	72 ***^/^°	<0.0001
	(72–77)	(70–75)	(70–75)	
@SD-AL2-MO	15.8	17.9 ***	18.6 ***^/^°	<0.0001
	(14.9–16.8)	(16.7–19.4)	(17.4–19.6)	
@MN-UMALS-EO	214	212	211 ***^/^°	<0.0001
	(210–217)	(207–217)	(206–215)	

The Kruskal–Wallis test and Dunn’s post hoc tests were employed for multiple group comparisons. ** *p* < 0.01, *** *p* < 0.001 versus controls; ° *p* < 0.05, °° *p* < 0.01 versus COVID.

**Table 4:** Performance of the logistic regression using 17 parameters (11 cellular population data), the haemoglobin level, the haematocrit, the red cell distribution width, and 3 parameters from the CytoDiff™ (the absolute count of B-lymphocytes and DC16pos monocytes and the ratio of CD16^pos^ monocytes/total monocytes) to discriminate patients with COVID-19 from patients with influenza.

Cut-Off	True Negatives	True Positives	False Negatives	False Positives	Sensitivity (%)	Specificity (%)	Efficiency (%)
0.4	17	233	5	36	97.7	32.1	85.9
0.5	23	229	9	30	96.2	43.4	86.6
0.6	28	218	20	25	91.6	52.8	84.5

## Data Availability

Data presented in this study are available upon request from the corresponding author.

## Supplementary Materials

The following supporting information can be downloaded at: https://www.mdpi.com/article/10.3390/jcm13010186/s1, Table S1: Comparison of the cellular population data (CPD) of 178 controls (C) with CPD of 349 patients with proven COVID-19, 102 patients with the flu, and 24 patients with RSV infection.
