# Supplementary material for: Can Haematological Parameters Discriminate COVID-19 from Influenza?
Source: J Clin Med. 2023 Dec 28;13(1):186. doi: 10.3390/jcm13010186 (PMC10780240; doi:10.3390/jcm13010186)
Supplement: Supplementary file 1 [file jcm-13-00186-s001.zip › jcm-2760148-supplementary.pdf]

Table S1. Comparison of the cellular population data (CPD) of 178 controls (C) with CPD of 349 patients with proven COVID-19, 102 patients with the flu, and 24 patients with RSV infection.

	Controls	SARS-CoV-2	Influenza Virus	*p*
@MN-V-NE	147 (144 – 152)	149** (145 – 155)	152***/°° (148 – 156)	< 0.0001
@SD-V-NE	17.1 (16.3 – 17.8)	17.8*** (16.9 – 19)	18.1***/° (17.3 – 19.1)	< 0.0001
@MN-C-NE	147 (145 – 148)	146* (144 – 148)	146* (144 – 148)	0.0145
@SD-C-NE	4.5 (4.2 – 4.9)	4.8*** (4.4 – 5.2)	4.9***/° (4.6 – 5.3)	< 0.0001
@MN-MALS-NE	140 (136 – 143)	140 (135 – 143)	140 (135 – 144)	0.6096
@SD-MALS-NE	10.1 (9.6 – 11.1)	10.3 (9.5 – 11.4)	10.5 (9.6 – 11.5)	0.5449
@MN-UMALS-NE	138 (135 – 141)	138 (134 – 143)	139 (135 – 145)	0.1232
@SD-UMALS-NE	10.7 (10.3 – 11.2)	10.8 (10.3 – 11.5)	10.8 (10.2 – 11.7)	0.4322
@MN-LMALS-NE	140 (133 – 144)	138 (131 – 144)	137 (131 – 144)	0.2380
@SD-LMALS-NE	13.1 (11.9 – 15.1)	13.3 (12.1 – 15.4)	14.1 (12.4 – 15.7)	0.1024
@MN-LALS-NE	177 (161 – 199)	176 (159 – 198)	172 (160 – 197)	0.6096
@SD-LALS-NE	30.9 (27.8 – 36.0)	31.5 (28.9 – 36.6)	32.4 (28.9 – 37.7)	0.2858
@MN-AL2-NE	142 (138 – 145)	142 (137 – 147)	143** (139 – 148)	0.0078
@SD-AL2-NE	11.0 (10.1 – 12.0)	11.8*** (10.8 – 12.9)	12.0*** (11.2 – 13.7)	< 0.0001
@MN-V-LY	87 (84 – 89)	91*** (88 – 95)	91*** (88 – 94)	< 0.0001
@SD-V-LY	13.6 (13.1 – 14.4)	15.3*** (13.9 – 17.0)	15.4*** (14.5 – 16.7)	< 0.0001
@MN-C-LY	114 (113 – 116)	115** (113 – 117)	115** (113 – 117)	0.0034
@SD-C-LY	7.3 (6.7 – 7.9)	7.7*** (6.8 – 8.9)	7.9*** (6.9 – 9.0)	0.0002
@MN-MALS-LY	70 (67 – 73)	67*** (63 – 71)	67*** (63 – 70)	< 0.0001
@SD-MALS-LY	16.1 (15.3 – 16.9)	17.3*** (16.1 – 18.7)	17.6*** (16.4 – 18.9)	< 0.0001
@MN-UMALS-LY	70 (66 – 74)	67*** (59 – 73)	67*** (59 – 71)	< 0.0001
@SD-UMALS-LY	20.0 (18.9 – 21.0)	21.7*** (19.8 – 23.6)	21.9*** (20.0 – 23.7)	< 0.0001
@MN-LMALS-LY	66 (63 – 70)	63*** (61 – 65)	63*** (61 – 66)	< 0.0001
@SD-LMALS-LY	18.5 (17.5 – 19.4)	19.5*** (18.1 – 20.6)	19.8*** (18.5 – 20.8)	< 0.0001
@MN-LALS-LY	41 (37 – 44)	42*** (39 – 46)	43*** (40 – 46)	< 0.0001
@SD-LALS-LY	11.2 (9.9 – 12.5)	12.3*** (10.9 – 14.1)	12.7*** (10.9 – 14.6)	< 0.0001

@MN-AL2-LY	72 (68 – 75)	76*** (72 – 80)	77*** (73 – 81)	< 0.0001
@SD-AL2-LY	12.7 (12.3 – 13.2)	13.2*** (12.6 – 14.0)	13.5*** (12.7 – 14.1)	< 0.0001
@MN-V-MO	176 (172 – 179)	183*** (177 – 191)	187***/°° (181 – 193)	< 0.0001
@SD-V-MO	18.8 (17.7 – 19.6)	23.7*** (21.5 – 25.3)	24.4***/°° (22.8 – 26.1)	< 0.0001
@MN-C-MO	123 (122 – 124)	124** (122 – 126)	121** (122 – 126)	0.0019
@SD-C-MO	4.9 (4.5 – 5.4)	5.1** (4.7 – 5.7)	5.1 (4.6 – 5.6)	0.0080
@MN-MALS-MO	88 (86 – 90)	86*** (83 – 89)	85***/° (82 – 88)	< 0.0001
@SD-MALS-MO	10.6 (9.9 – 11.3)	11.6*** (10.8 – 12.5)	11.7*** (10.8 – 12.6)	< 0.0001
@MN-UMALS-MO	99 (96 – 101)	96*** (92 – 99)	95*** (90 – 99)	< 0.0001
@SD-UMALS-MO	12.0 (11.2 – 12.7)	12.7*** (11.6 – 14.0)	12.7*** (11.6 – 13.8)	< 0.0001
@MN-LMALS-MO	75 (72 – 77)	73*** (70 – 75)	72***/° (70 – 75)	< 0.0001
@SD-LMALS-MO	13.6 (12.8 -14.7)	15.5*** (14.4 -16.7)	15.8*** (14.7 -16.8)	< 0.0001
@MN-LALS-MO	90 (80 – 102)	84*** (72 – 96)	83** (73 – 98)	< 0.0001
@SD-LALS-MO	27.6 (22.2 – 32.1)	31.2*** (26.7 – 36.3)	31.8*** (27.7 – 37.2)	< 0.0001
@MN-AL2-MO	128 (121 – 132)	132*** (126 – 139)	134*** (128 – 142)	< 0.0001
@SD-AL2-MO	15.8 (14.9 – 16.8)	17.9*** (16.7 – 19.4)	18.6***/° (17.4 – 19.6)	< 0.0001
@MN-V-EO	160 (156 – 165)	155*** (149 – 160)	157*** (152 – 161)	< 0.0001
@SD-V-EO	16.6 (15.5 – 17.8)	15.3*** (13.2 – 17.4)	15.6* (12.5 – 18.2)	< 0.0001
@MN-C-EO	148 (146 – 150)	150*** (147 – 153)	150*** (148 – 153)	< 0.0001
@SD-C-EO	4.0 (3.7 – 4.5)	3.8** (3.3 – 4.7)	3.9 (3.1 – 5.4)	0.0084
@MN-MALS-EO	202 (199 – 205)	202 (198 – 206)	201 (197 – 204)	0.1457
@SD-MALS-EO	8.4 (7.7 – 9.0)	8.3 (7.1 – 9.5)	8.1 (7.1 – 9.4)	0.7786
@MN-UMALS-EO	214 (210 – 217)	212 (207 – 217)	211***/° (206 – 215)	0.0021
@SD-UMALS-EO	9.2 (8.6 – 10.0)	9.2 (7..9 – 10.9)	9.5 (8.2 – 11.4)	0.6653
@MN-LMALS-EO	188 (185 – 192)	189 (184 – 193)	189 (184 – 193)	0.4030
@SD-LMALS-EO	10.6 (9.6 – 11.6)	10.2** (8.5 – 11.6)	10.0*** (8.1 – 10.8)	< 0.0001
@MN-LALS-EO	176 (160 – 189)	167** (153 – 186)	177 (154 – 191)	< 0.0001
@SD-LALS-EO	43.5	41.2***	42.7	< 0.0001

	(40.8 – 46.1)	(34.4 – 45.7)	(37.1 – 46.8)	
@MN-AL2-EO	121<br>(118 – 123)	119**<br>(114 – 124)	119*<br>(113 – 124)	0.8927
@SD-AL2-EO	9.6<br>(8.7 – 10.5)	9.5<br>(7.9 – 10.9)	9.3<br>(7.9 – 11.3)	0.9376

* $p < 0.0$, ** $p < 0.01$, *** $p < 0.001$ versus controls; ° $p < 0.05$, °° $p < 0.01$ versus COVID-19; the Kruskal–Wallis test and Dunn's post hoc tests were employed for multiple group comparisons.
